# mRNA nanomedicine: Design and recent applications

**DOI:** 10.1002/EXP.20210217

**Published:** 2022-09-19

**Authors:** Luke J. Kubiatowicz, Animesh Mohapatra, Nishta Krishnan, Ronnie H. Fang, Liangfang Zhang

**Affiliations:** ^1^ Department of NanoEngineering, Chemical Engineering Program, and Moores Cancer Center University of California San Diego La Jolla California USA

**Keywords:** biomimetic nanomedicine, cancer immunotherapy, gene therapy, lipid nanoparticle, viral vaccine

## Abstract

The rational design and application of mRNA‐based medicine have recently yielded some key successes in the clinical management of human diseases. mRNA technology allows for the facile and direct production of proteins in vivo, thus circumventing the need for lengthy drug development cycles and complex production workflows. As such, mRNA formulations can significantly improve upon the biological therapies that have become commonplace in modern medicine. Despite its many advantages, mRNA is inherently fragile and has specific delivery requirements. Leveraging the engineering flexibility of nanobiotechnology, mRNA payloads can be incorporated into nanoformulations such that they do not invoke unwanted immune responses, are targeted to tissues of interest, and can be delivered to the cytosol, resulting in improved safety while enhancing bioactivity. With the rapidly evolving landscape of nanomedicine, novel technologies that are under development have the potential to further improve the clinical utility of mRNA medicine. This review covers the design principles relevant to engineering mRNA‐based nanomedicine platforms. It also details the current research on mRNA nanoformulations for addressing viral infections, cancers, and genetic diseases. Given the trends in the field, future mRNA‐based nanomedicines have the potential to change how many types of diseases are managed in the clinic.

## Graphical abstract

mRNA nanomedicines have shown considerable promise as prophylactic and therapeutic agents given their capacity to enable the in situ production of any protein. In this review, we discuss the design of nanoparticle‐based platforms for mRNA delivery, as well as their recent application to viral infections, cancers, and genetic diseases.

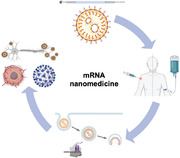

## 1 INTRODUCTION

The concept of leveraging mRNA for prophylactic and therapeutic applications has been under development for the past several decades.^[^ ^1^ ^]^ Despite the shortcomings of mRNA when administered by itself, the theoretical premise that any protein can be produced in situ for elongated periods of time has driven researchers across many disciplines to work toward realizing its potential.^[^ ^2^ ^]^ To this end, most of the focus has been placed on the design of mRNA with improved stability, immunogenicity, and purity, as well as incorporation with delivery vehicles capable of improving transfection efficiency in vivo. The application of nanoparticles for the delivery of mRNA was a natural choice given their established success in other areas of medicine.^[^ ^3^, ^4^, ^5^ ^]^ Specifically for mRNA medicine, nanoparticles offer four primary advantages: protection from premature degradation, prolonged serum residence time, site‐specific targeting, and cytosolic delivery. To access these advantages, mRNA must be incorporated with nanoparticles in a reliable and stable manner. The majority of current nanoformulations leverage positively charged components to electrostatically complex with the negatively charged mRNA backbone,^[^ ^6^, ^7^ ^]^ a strategy that has worked well for other nucleic acid‐based payloads.^[^ ^8^, ^9^ ^]^ Other design aspects, such as the use of various surface functionalization approaches to prolong circulation and facilitate active targeting, have been readily adapted from both well‐established and emerging technologies.^[^ ^3^, ^10^, ^11^ ^]^ Perhaps the most challenging aspect of mRNA delivery is the requirement for localization to the cytosol, where protein translation occurs; encouragingly, many potential solutions based on variations of the proton‐sponge effect, membrane destabilization, and ligand‐based fusion are in development.^[^ ^12^, ^13^, ^14^, ^15^ ^]^

**FIGURE 1:**
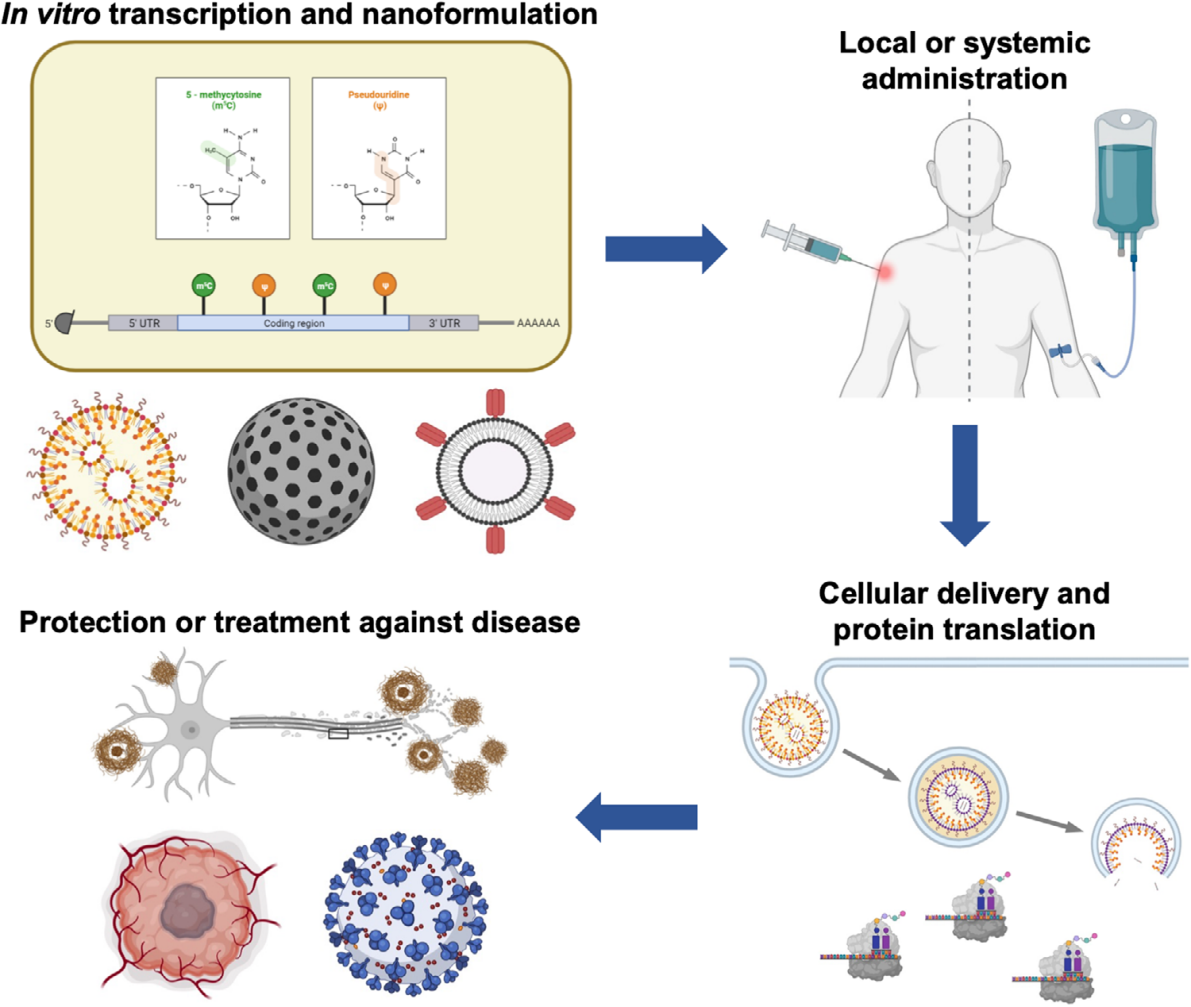
mRNA nanomedicine. mRNA synthesized by in vitro transcription can be formulated using various nanoparticle‐based platforms. The resulting nanoformulations can be administered via local or systemic routes, depending on the desired application. Upon uptake, the mRNA payloads must localize to the cytosolic compartment for translation into proteins. mRNA nanomedicine is currently being explored for the prevention or treatment of many different conditions, including viral infections, cancers, and genetic diseases. Created with BioRender

Research on mRNA nanodelivery over the past several decades has resulted in the development of prophylactics and therapeutics that are actively being investigated against a broad spectrum of disease states (Figure 1). Currently, there are a significant number of mRNA‐based interventions that are being evaluated in clinical trials (Table 1). Most notably, the application of mRNA nanomedicine against viral infection has been particularly successful, resulting in vaccination strategies that have proven highly effective in combating the coronavirus disease of 2019 (COVID‐19) pandemic.^[^ ^16^, ^17^ ^]^ Against pathogenic diseases, nanoparticles deliver mRNAs encoding for antigens or antibodies that are used to either train or assist the immune system in clearing infections. mRNA technology has also been widely explored for cancer immunotherapies to mobilize the immune system against immunologically “cold” tumors.^[^ ^18^, ^19^ ^]^ In addition, mRNA nanomedicine has unique potential as a therapeutic approach for genetic diseases;^[^ ^15^, ^20^, ^21^ ^]^ a single injection of an mRNA encoding for a replacement protein can sustain physiologically relevant concentrations for an extended period, thereby increasing therapeutic effectiveness and patient compliance. In this review, we first provide a brief discussion on how mRNA can be leveraged for medical applications, followed by an overview of how nanoparticles have been utilized for mRNA delivery. Finally, various applications of mRNA medicine, including against viral infections, cancers, and genetic diseases, are covered in detail.

**TABLE 1:** Representative list of clinical trials in the United States investigating new mRNA‐based interventions

mRNA intervention(s)	Condition	Administration route	Identifier	Phase
1. SAM‐LNP‐S 2. SAM‐LNP‐S‐TCE	COVID‐19 infection	Intramuscular	NCT04776317	Phase 1
mRNA‐1273.351	COVID‐19 infection	Intramuscular	NCT04785144	Phase 1
mRNA‐1283	COVID‐19 infection	Intramuscular	NCT04813796	Phase 1
CV2CoV	COVID‐19 infection	Intramuscular	NCT05260437	Phase 1
mRNA‐1189	Epstein–Barr virus infection	Intramuscular	NCT05164094	Phase 1
1. mRNA‐1644 2. mRNA‐1644v2‐Core	HIV infection	Intramuscular	NCT05001373	Phase 1
1. BG505 MD39.3 mRNA 2. BG505 MD39.3 gp151 mRNA 3. BG505 MD39.3 gp151 CD4KO mRNA	HIV infection	Intramuscular	NCT05217641	Phase 1
mRNA‐1653	Human metapneumovirus and human parainfluenza infection	Intramuscular	NCT04144348	Phase 1
Liposomal autologous total tumor mRNA, pp65 mRNA, and LAMP mRNA	Adult glioblastoma	Intravenous	NCT04573140	Phase 1
mRNA‐2752	Various cancers	Intratumoral	NCT03739931	Phase 1
mRNA‐5671/V941	Various cancers	Intramuscular	NCT03948763	Phase 1
mRNA‐3745	Glycogen storage disease	Intravenous	NCT05095727	Phase 1
ARCT‐810	Ornithine transcarbamylase deficiency	Intravenous	NCT04442347	Phase 1
mRNA‐1010	Seasonal influenza infection	Intramuscular	NCT04956575	Phase 1/2
1. mRNA‐1020 2. mRNA‐1030	Seasonal influenza infection	Intramuscular	NCT05333289	Phase 1/2
BNT112	Prostate cancer	Intravenous	NCT04382898	Phase 1/2
mRNA‐2416	Various cancers	Intratumoral	NCT03323398	Phase 1/2
BNT141	Various cancers	Intravenous	NCT04683939	Phase 1/2
UX053	Glycogen storage disease type III	Intravenous	NCT04990388	Phase 1/2
mRNA‐3927	Propionic acidemia	Intravenous	NCT04159103	Phase 1/2
mRNA‐1893	Zika virus infection	Intramuscular	NCT04917861	Phase 2
mRNA‐4157	Melanoma	Intramuscular	NCT03897881	Phase 2
mRNA‐1647	Cytomegalovirus infection	Intramuscular	NCT05085366	Phase 3
mRNA‐1345	Respiratory syncytial virus infection	Intramuscular	NCT05330975	Phase 3

## 2 mRNA‐BASED MEDICINE

Proteins are incredibly diverse and serve as essential biological building blocks that account for a wide range of cellular functions. As such, a considerable number of disease states arise from some type of protein dysfunction or dysregulation. Additionally, proteins from foreign organisms serve as antigenic cues against which adaptive immune responses are mounted, thus enabling hosts to combat infectious diseases. Using this knowledge, a large number of traditional vaccines and therapeutics have relied on the exogenous administration of either naturally occurring or engineered proteins.^[^ ^22^, ^23^ ^]^ Despite many clinical successes, this strategy suffers from several drawbacks, including the need to optimize manufacturing on a per protein basis and the difficulty in maintaining physiologically relevant concentrations in patients. More recently, researchers have turned to nucleic acid‐based strategies, which are beginning to show promise in clinical trials.^[^ ^16^, ^17^, ^24^, ^25^, ^26^ ^]^ Notably, mRNA‐based solutions, with their streamlined production workflows and design flexibility, have been viewed as highly attractive alternatives to protein‐based platforms. The basic role of mRNA is to effectuate specific protein expression instructions from DNA, which is confined to the nucleus of a cell. Upon transcription, mRNA travels to the cytoplasm, where it works with translation machinery such as ribosomal subunits and tRNA to produce its encoded protein. mRNA was first discovered in the early 1960s, and it did not take long before researchers observed that exogenous mRNA could induce protein expression in the cells that internalized it,^[^ ^1^ ^]^ prompting further investigation into how this phenomenon could be used to address human diseases.

In general, an mRNA molecule is sequentially comprised of a 5′ cap, 5′ untranslated region (UTR), coding sequence, 3′ UTR, and polyadenylation (polyA) tail.^[^ ^27^ ^]^ When made synthetically, each section of an mRNA molecule can be engineered to fit the desired application, offering design flexibility in both the encoded protein product and how the product is translated.^[^ ^2^, ^6^, ^27^, ^28^, ^29^ ^]^ The 5′ cap generally consists of a 7‐methylguanosine or synthetic analog that protects the mRNA from degradation, impacts translation efficiency, and alters immunogenicity. The sequence of the UTR regions can be optimized to include motifs that regulate interactions with microRNAs as well as proteins that are involved in the translation process. The coding sequence can be improved by employing codons that are more commonly occurring. Lastly, the polyA tail length can be modulated to control the stability of the mRNA. Synthetic mRNA is commonly produced through the process of in vitro transcription (IVT), which utilizes a DNA template, a mix of mRNA nucleotides, a 5′ capping reagent, and an RNA polymerase to produce mRNA strands in a one‐pot synthesis that mimics the natural cellular transcription process.^[^ ^30^ ^]^ DNase is then generally applied to remove the template, followed by a polyA tailing step. Finally, some form of purification such as fast performance liquid chromatography or LiCl precipitation is used to remove unreacted components and/or double stranded RNA contaminants,^[^ ^2^ ^]^ after which the mRNA molecule is ready for use.

The theoretical ability of mRNA technology to produce any protein of interest has been a significant driver of its continued advancement in the biomedical field. Compared with DNA‐based gene delivery platforms, mRNAs also offer the benefit of being further downstream in the protein expression process, thus mitigating concerns of genomic integration.^[^ ^2^ ^]^ Moreover, the accessible nature of genetic sequencing and relative ease of manufacturing enable mRNA‐based strategies to be rapidly developed and deployed, which has proven to be highly beneficial in the response against COVID‐19.^[^ ^31^ ^]^ However, mRNAs have their own set of weaknesses that must be mitigated before their full potential can be realized. For example, mRNA that is produced by IVT using more traditional nucleosides can exhibit decreased translation and is more prone to activating an immune response through interactions such as with Toll‐like receptors.^[^ ^32^ ^]^ Researchers have since discovered that mRNA produced using chemically modified uridine and/or cytidine can address some of these issues,^[^ ^2^ ^]^ and further development along these lines may yield even more significant improvements. Additionally, mRNA suffers from poor stability and ineffective cellular uptake, hindering its transfection efficiency without the help of a delivery mechanism.^[^ ^33^ ^]^ Nanobiotechnology offers a potent solution to this problem, and many promising strategies have been developed in recent years.

## 3 NANOPARTICLE DELIVERY OF mRNA

Nanomedicine is a highly interdisciplinary field that aims to increase the safety and efficacy of traditional medicines through the unique properties of nanoparticles.^[^ ^34^, ^35^, ^36^ ^]^ One advantage is the ability to increase blood circulation time, which is particularly important given the low solubility and poor stability of many drug molecules.^[^ ^11^, ^37^, ^38^ ^]^ Poor pharmacokinetics and biodistribution can result from several factors, including drug hydrophobicity and the lack of a targeting modality to direct treatments to the desired site of action. Furthermore, rapid clearance by the innate immune system or various organs like the kidneys and liver further decreases circulation time, resulting in a sizable proportion of the therapeutics being destroyed or misdirected.^[^ ^39^, ^40^, ^41^ ^]^ Prolonging the blood residence of drug molecules using nanocarriers can improve bioactivity while also limiting toxicity to healthy cells.^[^ ^11^, ^42^, ^43^, ^44^, ^45^ ^]^ Through optimized pharmacokinetics, nanodrugs have a higher chance of being utilized at their intended site of action, thus leading to improved bioavailability.^[^ ^46^, ^47^ ^]^ Passive targeting relies on a nanoparticle's structural properties, including its size, shape, and charge, to guide accumulation and cargo release.^[^ ^48^, ^49^, ^50^, ^51^ ^]^ Longer circulation times are particularly useful for nanoparticle‐based cancer therapeutics, as they can exploit the enhanced permeability and retention effect caused by the leaky vasculature that is characteristic of many tumors.^[^ ^52^, ^53^ ^]^

In contrast to the passive effects of prolonged circulation, the active targeting of nanoparticles aims to improve treatment efficacy by leveraging ligands that are specific to the desired site of action.^[^ ^54^, ^55^, ^56^ ^]^ This can also improve safety and reduce side effects by minimizing the exposure of healthy cells to the drug payload.^[^ ^57^ ^]^ Depending on the strategy that is used, nanoparticles can be targeted to specific organs, cell types, and even intracellular organelles.^[^ ^58^, ^59^, ^60^, ^61^ ^]^ Peptides and proteins are commonly used to target cells due to their ability to participate in high‐affinity ligand–receptor interactions.^[^ ^62^ ^]^ Alternate methods include the use of aptamers, which are a class of oligonucleotides, and small molecules such as folic acid.^[^ ^63^, ^64^ ^]^ Strategies for active targeting are most effectively employed when the targeted site contains a unique or overexpressed receptor whose ligand can be functionalized onto the exterior of a nanoparticle.^[^ ^57^, ^65^ ^]^ Active targeting oftentimes serves as a complement to passive targeting, and the presence of specific binding interactions can help to improve accumulation and retention at the target site as nanoparticles circulate through the body; the combined use of active and passive targeting is a widespread practice and maximizes the statistical probability of successful payload delivery.^[^ ^56^ ^]^

The ability of nanoparticle platforms to improve the efficacy and safety of therapeutics makes them a rational choice for the delivery of mRNA medicines. Despite its promise for preventing and treating various diseases, mRNA suffers from poor stability in vivo and the inability to efficiently enter cells.^[^ ^2^ ^]^ The administration of naked mRNA strands leaves them exposed to rapid degradation by ribonucleases in the serum and within target cells.^[^ ^66^, ^67^, ^68^ ^]^ Additionally, RNA molecules can be recognized by Toll‐like receptors in cells, leading to unwanted immune reactions that can compromise both safety and efficacy.^[^ ^66^, ^69^, ^70^ ^]^ Due to these issues, mRNA must be combined with a delivery vehicle that protects it from degradation and immune detection in a manner that retains potency.^[^ ^32^, ^71^, ^72^ ^]^ Lipid nanoparticles (LNPs) are one of the most widely adopted solutions;^[^ ^6^, ^73^ ^]^ they have also been used to formulate RNA interference therapeutics such as the FDA‐approved patisiran.^[^ ^74^ ^]^ LNPs are generally composed of cationic or ionizable lipids, phospholipid helpers, cholesterol, and polyethylene glycol (PEG)‐conjugated lipids.^[^ ^29^, ^75^, ^76^ ^]^ Cationic lipids were the initial choice for LNP fabrication since their positively charged head groups are capable of complexing with the negatively charged mRNA backbone while concurrently facilitating cytosolic delivery. However, the positive surface charge of these formulations generated biocompatibility concerns. As a result, the use of ionizable lipids in place of cationic lipids has become increasingly popular. The chemical structure of ionizable lipids allows them to acquire a positive charge when exposed to lower pH conditions, which is ideal for mRNA encapsulation and endosomal escape, while their neutral charge at physiological pH reduces unwanted interactions following administration.^[^ ^77^, ^78^, ^79^, ^80^ ^]^

The synthesis of LNP‐based mRNA formulations involves a self‐assembly process that is driven by hydrophobic and electrostatic interactions.^[^ ^6^, ^29^, ^78^, ^81^ ^]^ In general, the lipid components of the LNP are dissolved in an organic phase, while the mRNA is dissolved in an aqueous phase. The two phases are then mixed together to produce the final formulation, and this can be done via thin‐film hydration, microfluidics, or T‐junction mixing.^[^ ^6^, ^29^, ^81^ ^]^ Microfluidic‐based approaches are more commonly employed during research and development due to their ability to generate smaller LNPs with higher encapsulation and improved size distributions relative to thin‐film hydration. T‐junction mixing is more common for large‐scale manufacture due to easier compliance with good manufacturing practice (GMP) regulations. The scalability, efficacy, and practicality of LNPs for mRNA delivery have been well‐demonstrated during the COVID‐19 pandemic, as many of the leading vaccine formulations are based on this technology.^[^ ^16^ ^]^

Current efforts in nanomedicine research are seeking to improve upon LNPs and other traditional nanoparticle designs through the use of biomimicry.^[^ ^82^ ^]^ Biomimetic and bioinspired nanoparticle systems are designed by leveraging solutions from naturally occurring biological structures that have evolved to overcome specific challenges.^[^ ^83^ ^]^ The manner in which a nanoparticle takes advantage of biomimicry can vary greatly. For example, red blood cells (RBCs) are known to circulate within the body for prolonged periods of time, thus leading researchers to design nanoparticles that mimic RBCs in terms of their form, composition, and function.^[^ ^84^ ^]^ It is also possible to design a nanosystem that functionally resembles something found in nature but is made entirely of artificial materials. This can be exemplified by filomicelles, which are hydrophobic, rod‐like micelles made from polymeric nanoparticles that utilize their high aspect ratio for prolonged circulation times and better drug loading.^[^ ^85^ ^]^ These structures mimic the tobacco mosaic virus, which has many of the same properties due to its own high aspect ratio structure.^[^ ^86^ ^]^ Additionally, biomimetic nanosystems can be created from biological nanomaterials such that they are compositionally similar to natural objects, an example being virus‐like particles (VLPs) for cargo delivery.^[^ ^87^, ^88^ ^]^

There has recently been significant interest in applying biomimetic nanoparticle platforms for mRNA delivery. In one case, Gram‐negative bacteria were genetically engineered to produce outer membrane vesicles (OMVs) displaying a lysosomal escape protein along with the L7Ae protein, which can bind to RNA containing the box C/D sequence.^[^ ^89^ ^]^ Following adsorption with mRNA encoding for a cancer antigen, the resulting formulation was utilized as a potent antitumor vaccine due to the natural adjuvant properties of the bacterial membrane. A similar system was developed in which implantable cells were genetically engineered to secrete exosomes internally packaged with catalase mRNA via the same L7Ae–box C/D mechanism.^[^ ^90^ ^]^ The exosomes were further modified for brain targeting and efficient cytosolic delivery. When the engineered cells were implanted into mice, significant reductions in neuroinflammation and neuronal death were achieved following exposure to a neurotoxin. Besides biological vesicles, VLPs have also been successfully engineered for mRNA delivery. For example, researchers found that expression of retrovirus‐like protein PEG10 leads to the production of VLPs capable of delivering mRNA flanked by PEG10 UTRs.^[^ ^91^ ^]^ Along similar lines, a lentiviral vector was engineered to package mRNA containing a structural MS2 stem loop via interactions with the MS2 coat protein.^[^ ^92^ ^]^

Cell membrane coating nanotechnology is another notable example of biomimicry that offers a unique and elegant approach to nanoparticle functionalization, enabling long circulation times and targeted delivery, among other advantages.^[^ ^93^, ^94^ ^]^ Cell membrane‐coated nanoparticles have a simple structure, consisting of a nanoparticulate core component that is surrounded by a layer of naturally derived cell membrane.^[^ ^95^, ^96^ ^]^ The cellular membrane is derived from live cells, endowing the nanoparticles with an array of lipids, carbohydrates, and proteins that enables them to exhibit cell‐mimicking properties such as camouflage from the immune system.^[^ ^93^ ^]^ Consequently, many cell membrane‐coated nanoparticle systems have been shown to circulate much more effectively than their purely synthetic counterparts.^[^ ^93^, ^96^, ^97^ ^]^ Cell membranes have been successfully harvested from mammalian cells, including RBCs, platelets, macrophages, cancer cells, and many others, as well as from various pathogen sources.^[^ ^3^, ^93^, ^97^, ^98^, ^99^, ^100^, ^101^ ^]^ The cell membrane coating can be further engineered for enhanced functionality through a variety of approaches such as lipid insertion, genetic alteration, and membrane hybridization.^[^ ^102^, ^103^, ^104^, ^105^ ^]^ The flexibility offered by cell membrane coating nanotechnology has proven useful across a wide range of applications, including drug delivery, immunotherapy, and detoxification, among others.^[^ ^99^, ^106^, ^107^, ^108^, ^109^ ^]^

**FIGURE 2:**
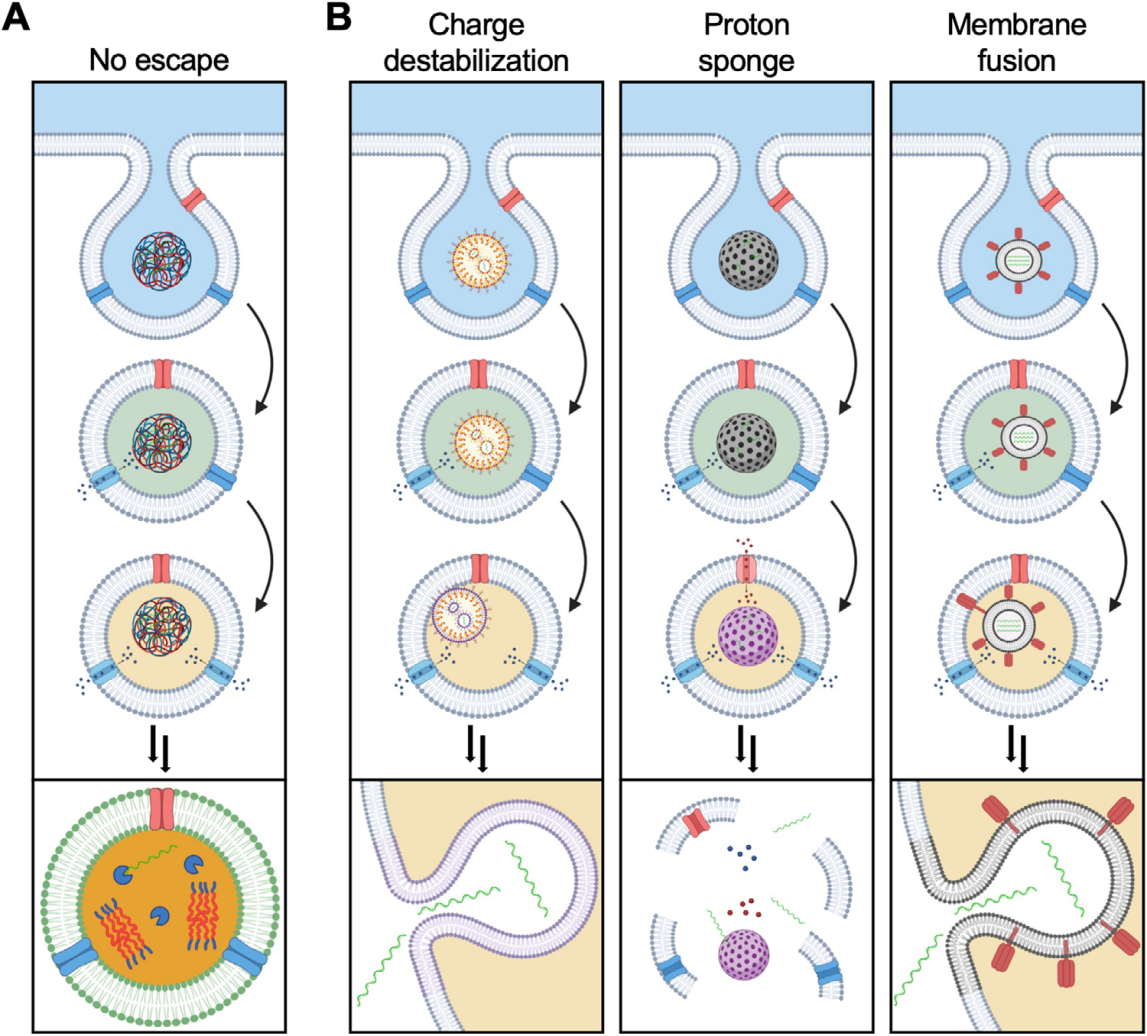
Nanoparticle‐mediated endosomal escape. (A) Under normal circumstances, nanoparticles are primarily taken up by cells via the endosomal pathway. In the absence of an escape mechanism, the nanoparticles and their contents will be subjected to endolysosomal degradation. (B) Several mechanisms for achieving endosomal escape have been explored, including charge‐based destabilization, the proton sponge effect, and direct membrane fusion. Following the escape, mRNA payloads can interact with the protein translation machinery located within the cytosolic compartment. Created with BioRender

Nanomedicine platforms can be engineered to more effectively achieve cytosolic localization, which is one of the most important considerations when it comes to the delivery of mRNA therapeutics (Figure 2).^[^ ^110^, ^111^, ^112^, ^113^, ^114^, ^115^, ^116^, ^117^, ^118^ ^]^ Nanoparticles must overcome several barriers before they can access the cytosol, where the cellular machinery responsible for protein translation resides.^[^ ^119^, ^120^, ^121^ ^]^ A majority of mRNA‐loaded nanoparticles will enter cells through endocytosis due to their size and receptor‐mediated targeting. Traditionally, nanoparticle entrapment within endosomes is a difficult challenge to overcome due to the harsh conditions within this subcellular compartment that will oftentimes destroy a nucleic acid payload before it can exert its biological activity.^[^ ^122^, ^123^, ^124^ ^]^ Endosomes form following the endocytosis of foreign objects and transition their interior from weakly acidic to moderately acidic, which in combination with various enzymes results in degradation of the internalized contents.^[^ ^12^, ^125^ ^]^ Eventually, the endosomes will fuse with a lysosome to facilitate further degradation.^[^ ^126^ ^]^

**FIGURE 3:**
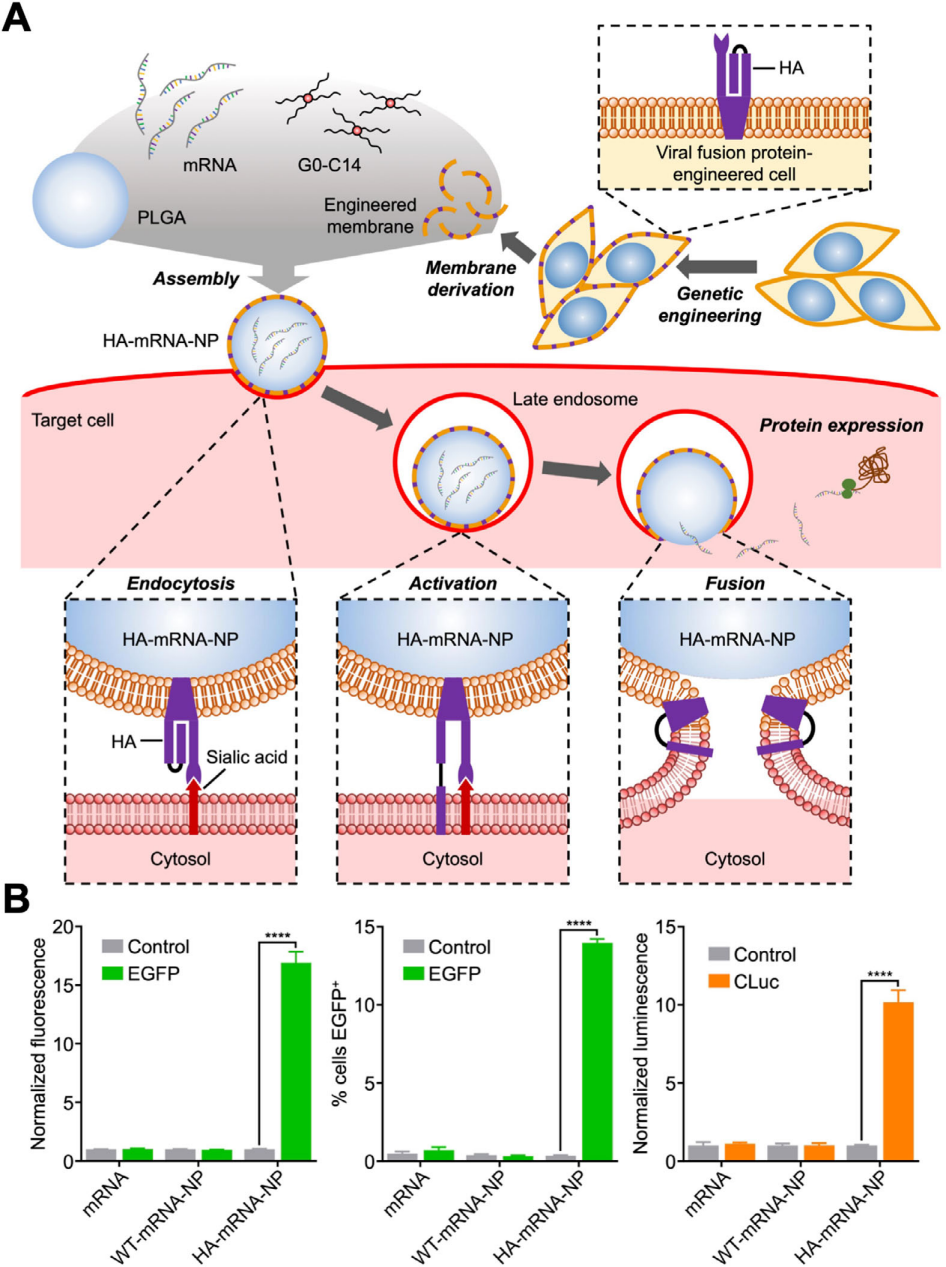
Virus‐mimicking cell membrane‐coated nanoparticles for the cytosolic delivery of mRNA. (A) mRNA is loaded into poly(lactic‐*co*‐glycolic acid) (PLGA) nanoparticles with the help of G0‐C14, followed by coating with cell membrane that expresses the influenza protein hemagglutinin (HA) to form HA‐mRNA‐NPs. The viral antigen on the nanoparticle surface enables escape from late endosomes following cellular uptake. (B) HA‐mRNA‐NPs loaded with mRNA encoding for either enhanced green fluorescent protein (EGFP) or *Cypridina* luciferase (CLuc) exhibit higher transfection efficiency compared to control nanoparticles coated with wild‐type cell membrane (WT‐mRNA‐NP) that lack HA expression. Reproduced with permission.^[^ ^13^ ^]^ Copyright 2021, Wiley‐VCH

With this in mind, researchers have put a strong focus on the development of methods to facilitate nanoparticle escape from endosomes. Solutions have included leveraging the proton sponge effect, membrane fusion, and endosomal escape ligands to achieve cytosolic delivery.^[^ ^12^ ^]^ For the proton sponge effect, nanoparticles are designed using components with pH buffering capabilities, thus hindering the ability of endosomes to decrease their internal pH.^[^ ^127^, ^128^, ^129^ ^]^ This results in a large number of protons being pumped into the endosomal lumen, which eventually leads to ion imbalances that cause osmotic stress and subsequent rupture of the endosome.^[^ ^130^, ^131^ ^]^ Most nanoparticles that rely on the proton sponge effect employ cationic polymers such as polyethylenimine and polyamidoamine, although some alternative materials have demonstrated considerable promise.^[^ ^132^, ^133^, ^134^, ^135^ ^]^ It is also possible to achieve escape by designing nanoparticles to destabilize the endosome through the formation of pores in the endosomal membrane during the early stages of endocytosis, thus allowing access to the cytosol.^[^ ^136^, ^137^, ^138^ ^]^ Another approach relies on direct interactions of the nanoparticle with the endosomal membrane after endosome formation.^[^ ^139^ ^]^ There are a variety of methods of accomplishing this, including the use of fusogenic peptides inspired by viruses, which often undergo a conformational change at lower pH values that allow for membrane disruption.^[^ ^140^, ^141^, ^142^, ^143^ ^]^ Recently, a unique biomimetic platform for cytosolic mRNA delivery based on cell membrane coating nanotechnology was reported (Figure 3).^[^ ^13^ ^]^ In the work, B16F10 cells were genetically engineered to express the influenza protein hemagglutinin (HA), which is known to facilitate endosomal escape at low pH. The cell membrane was then derived for coating onto mRNA‐loaded polymeric nanoparticle cores. It was demonstrated that the resulting nanoformulation could effectively achieve endosomal escape, leading to the successful expression of mRNA payloads encoding for enhanced green fluorescent protein and *Cypridina* luciferase. Compared with wild‐type membrane‐coated nanoparticle controls, the HA‐expressing formulation displayed enhanced mRNA transfection efficiency in vivo through multiple administration routes. Overall, future development of this novel platform and others could provide significant benefits to advance the field of mRNA nanomedicine.

## 4 APPLICATION OF mRNA NANOMEDICINE

### 4.1 Viral infection

Traditional viral vaccines rely on the safe and effective administration of whole (inactivated or attenuated) or partial (subunit) viruses as training tools for the immune system. However, these strategies are often slow in their development, making it hard to keep pace with newly emerging virus strains.^[^ ^2^ ^]^ In contrast, the facile payload interchangeability of mRNA‐based nanomedicine platforms can significantly streamline the development process. Optimal vaccine targets can be quickly discovered through genetic sequencing, rapidly yielding templates for subsequent large‐scale mRNA production. The rapid discovery process, synergistically paired with relatively inexpensive biomanufacturing costs for LNP formulations, have enabled mRNA vaccine candidates to reach clinical testing and receive regulatory authorization much faster than traditional vaccines.^[^ ^31^, ^144^ ^]^ This was exemplified by the recent development and deployment of the Pfizer‐BioNTech BNT162b2 and Moderna mRNA‐1273 mRNA vaccines to combat the COVID‐19 pandemic.^[^ ^16^, ^17^ ^]^ Both vaccines contain nucleoside‐modified mRNAs that induce the membrane‐bound expression of a perfusion‐stabilized, full‐length severe acute respiratory syndrome coronavirus 2 (SARS‐CoV‐2) spike protein. In each case, the mRNA vaccines were formulated using LNPs for intramuscular injection. The rapid development and potent efficacy of these vaccines will serve as a strong benchmark for the advancement of future mRNA‐based vaccines against a broad set of diseases. Despite the strong successes of these vaccines, the need for frozen storage and short‐term usability when thawed represent a barrier for widespread global distribution.^[^ ^145^ ^]^ Fortunately, strong efforts are being undertaken to overcome this challenge, and additional mRNA‐based nanovaccines against COVID‐19 are actively being developed.^[^ ^144^, ^146^, ^147^ ^]^

**FIGURE 4:**
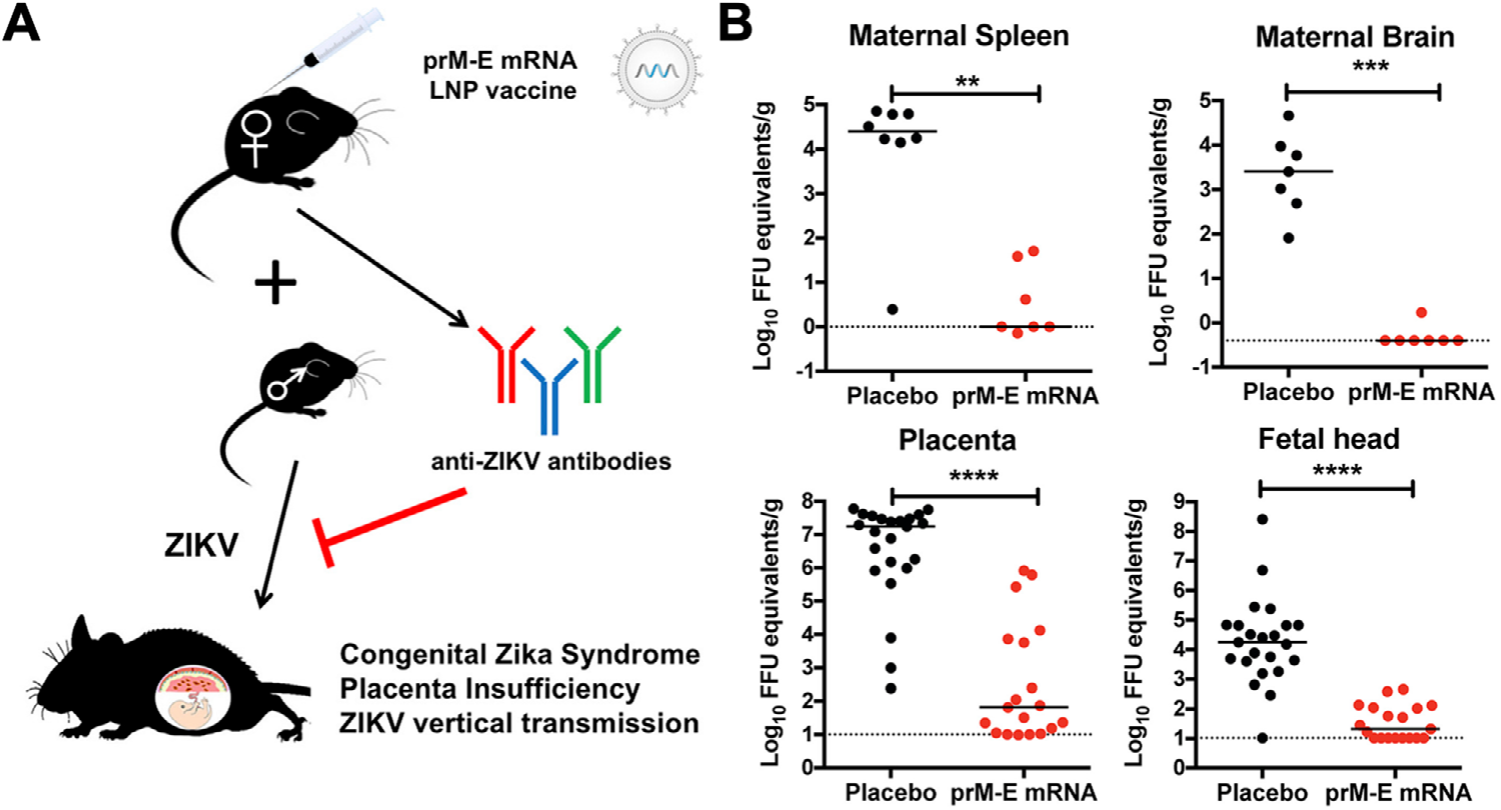
LNP mRNA vaccine against Zika virus (ZIKV)‐induced congenital disease. (A) An LNP vaccine encoding for the ZIKV pre‐membrane (prM) and envelope (E) proteins elicits anti‐ZIKV titers that can prevent congenital ZIKV syndrome. (B) Vaccination of female mice prior to pregnancy using LNPs loaded with prM and E mRNA following a prime plus boost strategy significantly protects pregnant mothers and their fetuses from the effects of ZIKV infection. Reproduced with permission.^[^ ^149^ ^]^ Copyright 2017, Elsevier

While the mRNA‐based vaccines against SARS‐CoV‐2 have garnered the most attention due to the global pandemic, there is a multitude of other promising formulations that are currently in development for other viral infections. For example, there have been several Zika virus (ZIKV) outbreaks over the past decade, prompting researchers to turn to mRNA‐based solutions. One group loaded an mRNA that encoded for the pre‐membrane and envelope glycoproteins of a 2013 ZIKV strain into an LNP for immunization.^[^ ^148^ ^]^ The co‐expression of both components enabled the assembly of subviral particles that were secreted by transfected cells to produce ZIKV‐specific immune responses. Mice vaccinated with a 30 µg dose developed virus‐specific T helper cell and neutralizing antibody responses, completely protecting against ZIKV challenges at 2 and 20 weeks post immunization. Furthermore, complete protection against a ZIKV challenge at 5 weeks post immunization was observed for non‐human primates that received the mRNA vaccines at 50 and 200 µg doses. ZIKV can have particularly devastating effects on the fetuses of expecting mothers who become infected. This prompted another group to test the protective efficacy of their LNP mRNA vaccine formulation in pregnant mice (Figure 4).^[^ ^149^ ^]^ A 10 µg dose was given to female mice using a prime plus boost schedule prior to mating, followed by a subsequent challenge with a heterologous ZIKV strain. The vaccinated group showed a significant decrease in ZIKV RNA detected from the maternal spleen, brain, placenta, and fetal head. The protective effects of the vaccine were further corroborated by data collected from mice vaccinated during pregnancy and then challenged with ZIKV. There has been some concern surrounding the potential for mRNA‐based vaccines to elicit antibody‐dependent enhancement, thus worsening the disease caused by mutated or similar viruses. One group sought to address this potential problem by mutating the ZIKV pre‐membrane and envelope glycoprotein mRNA.^[^^]^ Despite destruction of the highly conserved fusion‐loop epitope of the ZIKV envelope protein, protective neutralizing antibody titers were still elicited. Mice receiving the pooled serum of other mice immunized with the mutated mRNA vaccine showed greater than 80% survival against a dengue virus challenge. This same challenge was lethal to all mice receiving the pooled serum of other mice immunized with the non‐mutated mRNA vaccine or a dose of monoclonal antibodies targeting ZIKV.

**FIGURE 5:**
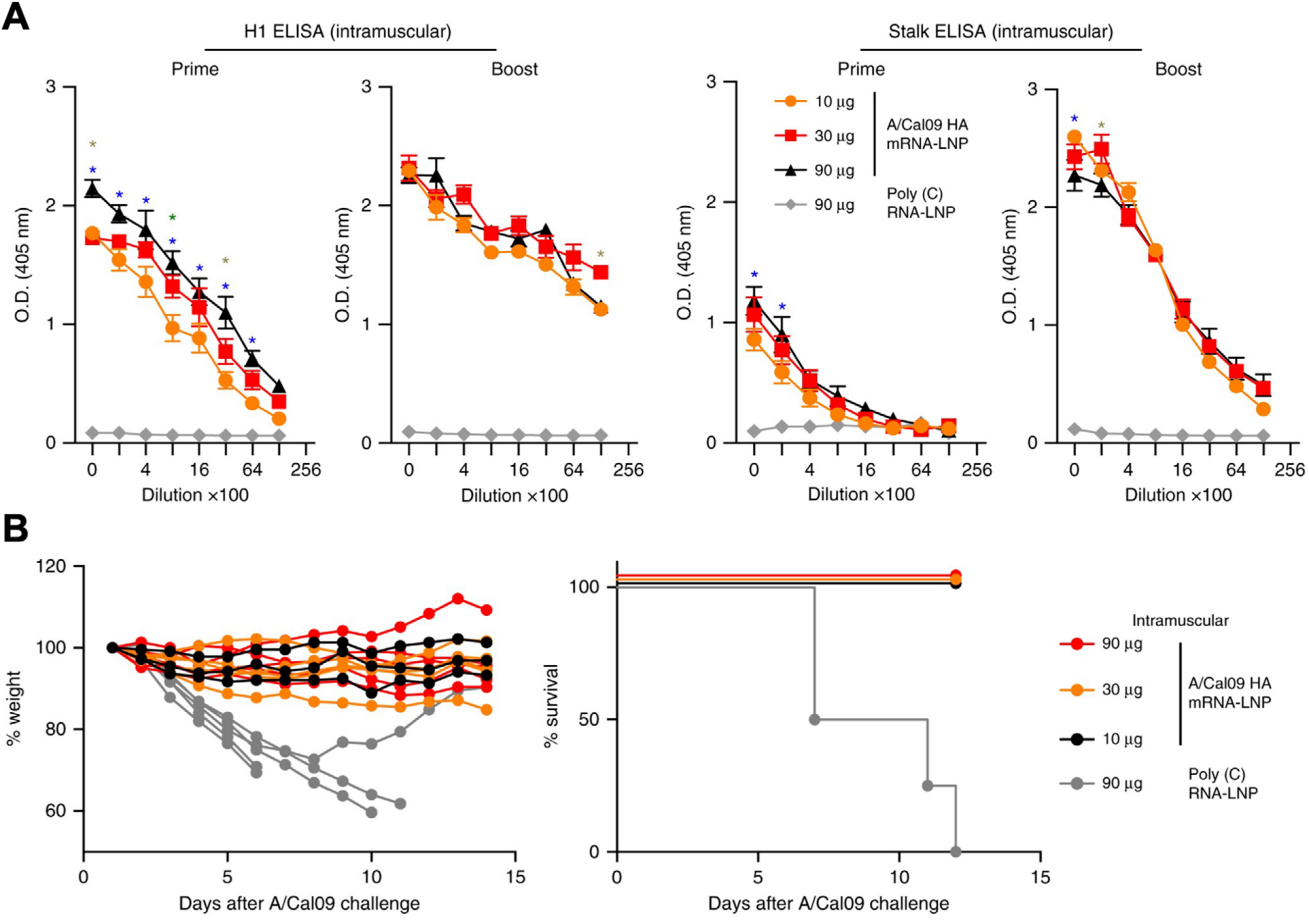
LNP mRNA vaccine against influenza targeting hemagglutinin (HA). (A) Mice vaccinated with A/California/07/2009 HA mRNA‐LNPs develop antibody titers specific to both the HA head region and HA stalk region. (B) Immunized mice do not exhibit major fluctuations in body weight and are 100% protected against an otherwise lethal A/California/07/2009 challenge. Reproduced with permission.^[^ ^151^ ^]^ Copyright 2018, Springer Nature

One potential criticism of mRNA‐based vaccines relative to traditional immunization strategies is the focus on a limited number of antigens, which may limit their application to unique outbreaks and open the door for viruses to evade the induced immune responses through rapid mutations. However, this concern can be alleviated through proper target selection. In one case, a broad‐spectrum vaccine was developed using an LNP formulation delivering mRNA that encoded for the full‐length HA protein from the A/California/07/2009 H1N1 influenza strain (Figure 5).^[^ ^151^ ^]^ HA can be split into a head region that is variable by strain and a stalk region that is conserved. Successful head and stalk antibody titers were obtained in mice, rabbits, and ferrets immunized with the mRNA‐based HA vaccine. The potency of this strategy was highlighted by the broad‐scale protection that it offered against several influenza strains. Mice receiving a single 30 µg dose of the vaccine were completely protected against otherwise lethal challenges of A/California/07/2009 (homologous H1N1) and A/Puerto Rico/8/1934 (heterologous H1N1). Furthermore, a prime plus boost regimen with the same dose also offered complete protection against a lethal challenge with A/Vietnam/1203/04 (heterosubtypic H5N1), which is more antigenically distinct.

Besides traditional vaccination strategies that train the immune system against a foreign antigen, mRNA‐based nanomedicines can also be used to directly produce virus‐neutralizing antibodies. In one example, this strategy was effectively utilized for the development of a treatment for chikungunya virus (CHIKV).^[^ ^152^ ^]^ A potent monoclonal antibody, CHKV‐24, was identified, and its corresponding mRNA was formulated into an LNP. A 0.5 mg/kg mRNA dose was able to produce strong serum concentrations of CHKV‐24 and prophylactically afforded AG129 mice full protection against an otherwise lethal CHIKV challenge. Furthermore, viral titers were suppressed with mRNA doses as low as 0.02 mg/kg on day 3 post treatment and day 2 post challenge. Wild‐type C57BL/6 mice receiving the mRNA‐based antibody treatment had strong improvements in parameters such as foot swelling, viremia, and detectable CHIKV RNA. The translatability of this nanoformulation was also evaluated in non‐human primates. A 0.5 mg/kg dose of mRNA delivered intravenously was sufficient to produce a CHKV‐24 serum concentration as high as 35.9 µg/ml, and detectable levels were maintained throughout the course of a 600‐h study. Impressively, a 3 mg/kg mRNA dose administered on days 0 and 7 in non‐human primates induced detectable CHKV‐24 for several months, with a serum concentration of 2.9 µg/ml on day 90. Similar therapeutic strategies have also displayed success against other infections caused by influenza A virus, rabies virus, and human immunodeficiency virus 1.^[^ ^153^, ^154^, ^155^ ^]^

### 4.2 Cancer

**FIGURE 6:**
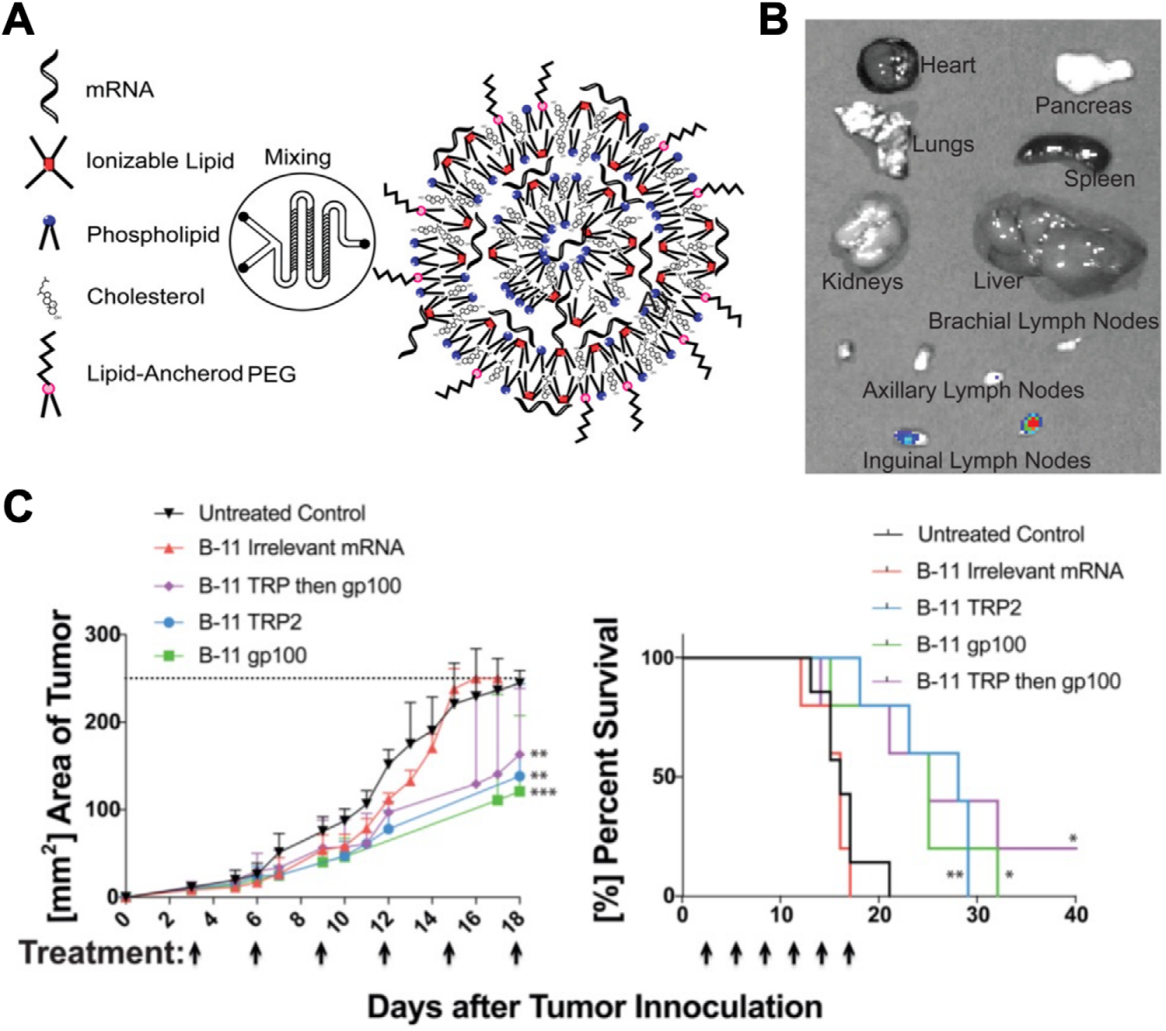
LNP mRNA vaccine against cancer. (A) mRNA‐loaded LNPs are synthesized by mixing the mRNA payload in an aqueous phase with the lipid components in an ethanol phase using a microfluidic device. At low pH, the ionizable lipid is positively charged and can complex with the negatively charged mRNA. (B) At 24 h after subcutaneous administration, the LNP formulation shows targeting of the draining lymph nodes. (C) Treatment of tumor‐bearing mice with LNPs carrying gp100 and TRP2 mRNA suppresses tumor growth and extends overall survival. Reproduced with permission.^[^ ^159^ ^]^ Copyright 2016, American Chemical Society

A wide range of anticancer vaccines and therapeutics are being developed utilizing mRNA technology, and many are already being evaluated in clinical trials.^[^ ^2^, ^156^ ^]^ Upon delivery, mRNA‐based anticancer nanovaccines are generally taken up by antigen‐presenting cells (APCs), after which the payload is translated into tumor antigen proteins.^[^ ^79^, ^157^ ^]^ These can then be processed into peptide epitopes and presented by major histocompatibility complex class I (MHC‐I) molecules to activate tumor‐specific CD8^+^ T cells, thereby promoting strong antitumor immunity.^[^ ^158^ ^]^ Along these lines, LNPs containing mRNA encoding for the tumor‐associated antigens gp100 and TRP2 were developed as a cancer vaccine against melanoma (Figure 6).^[^ ^159^ ^]^ Efficient targeting of the draining lymph nodes was observed, leading to the transfection of multiple immune cell subsets, including dendritic cells, macrophages, neutrophils, and B cells. The nanovaccine was able to slow tumor growth and extend overall survival past 40 days in a B16F10 melanoma model, whereas mice in control groups died within 21 days. In another notable example, RNA lipoplexes were developed and shown to specifically target dendritic cells upon systemic administration.^[^ ^160^ ^]^ By optimally adjusting the net charge of well‐known lipid carriers to be near‐neutral or slightly negative, efficient mRNA encapsulation and spleen targeting were achieved. The formulation conferred complete and long‐lasting protection against tumor growth in both B16F10 and CT26 tumor models, while all untreated mice died within 30 days of challenge. In a phase I clinical trial using RNA lipoplexes, over 75% of the trial subjects thus far have shown immune responses against at least one tumor‐associated antigen, as well as activation of antigen‐specific CD8^+^ T cells.^[^ ^161^ ^]^ Combination with programmed cell death protein 1 (PD‐1) checkpoint blockade further improved treatment efficacy, resulting in a 35% tumor regression rate. In recent years, additional efforts have been made to further improve LNP composition through the use of novel ionizable lipid‐like materials, as well as to explore the combination of mRNA nanovaccines with other immunotherapeutic modalities.^[^ ^162^, ^163^ ^]^

Besides LNPs, other platforms such as mesoporous silica nanoparticles and lipid–polymer hybrid nanoparticles have also demonstrated promise for augmenting mRNA‐based anticancer strategies.^[^ ^164^ ^]^ In one study, mesoporous silica nanoparticles carrying C16, a selective inhibitor of double‐stranded RNA‐dependent protein kinase, were delivered alongside free mRNA as a prophylactic treatment against thymic lymphoma.^[^ ^165^ ^]^ By co‐delivering C16, this platform was able to improve mRNA translation and prolong protein expression. In an in vivo mouse model, prophylactic vaccination with the formulation reduced tumor growth and resulted in 80% survival, compared to 0% survival in the unvaccinated control group. Lipid–polymer hybrid nanoparticles incorporate the advantages of polymeric nanoparticles with the advantages of liposomes into a single platform.^[^ ^166^, ^167^, ^168^ ^]^ In a proof‐of‐concept work, lipid‐enveloped pH‐responsive polymeric nanoparticles were formulated for the delivery of mRNA payloads.^[^ ^169^ ^]^ The platform consisted of a biodegradable poly(β‐amino ester) core, which was encapsulated by a phospholipid shell. The pH‐responsive core promoted endosomal disruption and escape, while the lipid surface enhanced biocompatibility. mRNA was efficiently adsorbed onto the surface of the positively charged nanoparticles via electrostatic interactions. The final nanoformulation displayed efficient uptake by dendritic cells in vitro and induced the expression of a reporter gene within 6 h after intranasal administration in an in vivo mouse model. In a later work that employed a similar lipid–polymer hybrid nanoparticle‐based strategy, functional delivery of mRNA to the lung endothelium and pulmonary immune cells was achieved after systemic administration.^[^ ^170^ ^]^ A different core–shell nanoplatform consisting of lipid‐coated calcium phosphate nanoparticles was functionalized with mannose to specifically target dendritic cells by binding to CD206.^[^ ^171^ ^]^ While the functionalized lipid exterior allowed for targeting, the inner core structure allowed for loading of nucleic acid payloads and endosomal escape after dissolution in low pH conditions. Delivery of these nanoparticles packaged with mRNA encoding for the melanoma‐associated antigen TRP2 and siRNA against programmed death‐ligand 1 (PD‐L1) resulted in significantly reduced tumor growth kinetics.

In addition to their success at training the endogenous immune system, mRNA‐based vaccines can also be used to enhance the efficacy of chimeric antigen receptor (CAR) T cell therapy.^[^ ^73^ ^]^ CARs are genetically engineered receptors that can redirect T cells to recognize and eliminate cells that express a specific target antigen.^[^ ^172^ ^]^ Against cancer, CAR T cells recognizing tumor antigens have been highly effective in some instances, particularly against B cell malignancies.^[^ ^173^ ^]^ In order to boost CAR T cell efficacy, an mRNA lipoplex formulation was designed to promote the expression of claudin 6, a newly identified CAR target for solid tumors, on host APCs.^[^ ^19^ ^]^ Upon intravenous administration of the nanoformulation, claudin 6 was expressed on splenic macrophages and dendritic cells, thus promoting the selective expansion of CAR T cells targeting the antigen. In a murine model of lung cancer positive for claudin 6, this strategy was highly effective at suppressing tumor growth, even at normally subtherapeutic CAR T cell dosages.

In an alternative approach to vaccination, mRNA‐based nanoplatforms have been used to induce a proinflammatory tumor microenvironment through the expression of cytokines and other immunostimulatory molecules.^[^ ^174^ ^]^ T cell‐stimulating cytokines have been employed clinically as powerful cancer treatments but can be limited by severe toxicity and adverse effects.^[^ ^175^ ^]^ Interleukin (IL)‐2 is a key cytokine in the differentiation, proliferation, and effector function of T cells.^[^ ^176^ ^]^ One LNP‐based strategy currently being explored leverages an extended half‐life variant of IL‐2.^[^ ^177^, ^178^ ^]^ In tumor‐bearing mice, systemic administration of the nanoformulation increased antitumor T cell responses, leading to strong control of tumor growth. Combination with PD‐1/PD‐L1 checkpoint blockade further increased T cell expansion and improved antitumor efficacy. In another example, a novel charge‐altering releasable transporter platform was used to deliver mRNA encoding for a combination of OX40L, CD80, and CD86.^[^ ^18^ ^]^ This approach allowed for impressive control of tumor growth in both A20 lymphoma and CT26 colon carcinoma models.

To reactivate tumor suppression and enhance antitumor immunity, a unique polymeric nanoparticle platform was developed for the delivery of mRNA encoding for PTEN, a tumor suppressor gene that is commonly lost in human cancers.^[^ ^179^ ^]^ The PTEN gene encodes for a dual phosphatase protein product that is important in the regulation of the cell cycle. With the loss of PTEN, cells can start to grow and divide uncontrollably. Nanoparticles based on methoxy PEG–poly(lactic‐*co*‐glycolic acid) were used to load mRNA complexed with a cationic lipid‐like material, 2‐epoxytetradecane‐modified generation 0 polyamidoamine. These mRNA‐loaded nanoparticles were able to restore the susceptibility of tumor cells to apoptosis and allowed for the release of damage‐associated molecular patterns, thus facilitating strong immune activation. Intravenous administration of the nanoformulation alongside intraperitoneal administration of PD‐1 checkpoint blockade resulted in impressive control of tumor growth, upregulation of inflammatory markers, and the increased presence of CD8^+^ T cells in a B16F10 model.

### 4.3 Genetic disease

**FIGURE 7:**
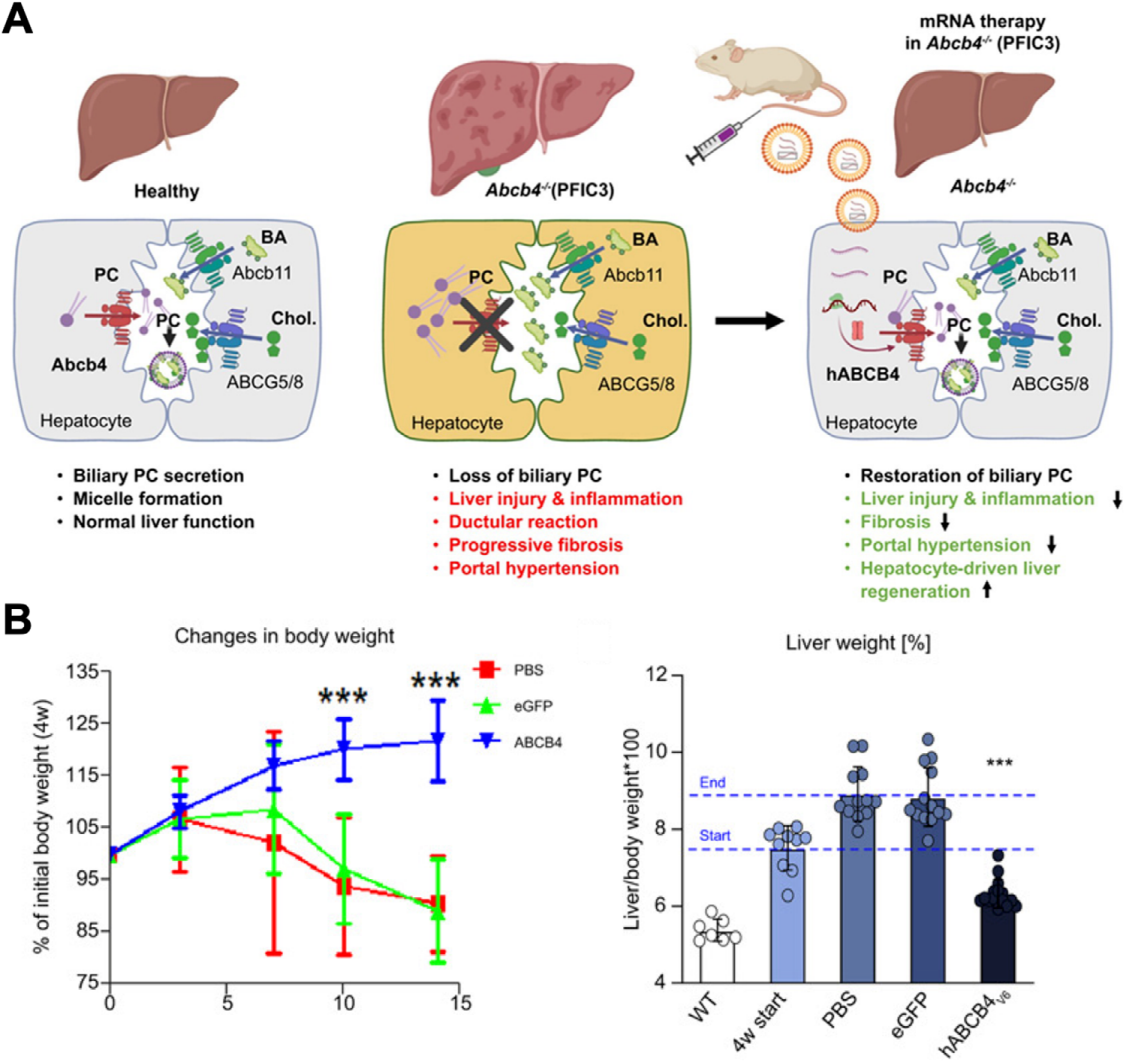
LNP mRNA treatment for progressive familial intrahepatic cholestasis type 3 (PFIC3). (A) Expression of ABCB4 using LNP mRNA nanoparticles can reverse the disease phenotype associated with PFIC3. (B) In an *Abcb4* knockout mouse model, treatment using LNPs loaded with mRNA encoding for ABCB4 reverses body weight loss and normalizes liver weight. Reproduced with permission.^[^ ^183^ ^]^ Copyright 2021, Elsevier

Genetic diseases can result in the loss of protein function, thereby necessitating protein replacement therapy.^[^ ^180^, ^181^ ^]^ Treatments must often be administered on a routine basis, thus representing a significant burden to patients. Therapeutic mRNA nanomedicines have the potential to circumvent this problem, as they can be leveraged for the in situ production of therapeutic proteins across a longer period of time.^[^ ^182^ ^]^ mRNA‐based protein replacement platforms have shown strong success in the treatment of liver diseases. This is largely because nanoparticles accumulate preferentially in the liver following systemic circulation. In one case, an mRNA‐loaded LNP formulation was developed for the treatment of progressive familial intrahepatic cholestasis type 3 (PFIC3), which occurs due to a defective *ABCB4* gene that normally encodes for a phosphatidylcholine (PC) transporter necessary for biliary micelle formation (Figure 7).^[^ ^183^ ^]^ After multiple injections of the nanoparticles formulated with codon‐optimized, nucleoside‐modified human *ABCB4* mRNA, significant improvements in body and liver weight, biliary PC concentrations, and other serum biomarkers were observed in BALB/c.Abcb4^–/–^ mice. Additionally, the treatments prevented liver fibrosis, as evidenced by improvements in collagen biomarkers, portal pressure, key profibrogenic molecule transcript levels, and matrix‐degrading enzyme levels. This liver‐targeting strategy was also found to be suitable for the treatment of hepatorenal tyrosinemia type 1, which is caused by mutations in the fumarylacetoacetate hydrolase (FAH) enzyme and can result in severe complications such as liver failure.^[^ ^184^ ^]^ In this case, a dendrimer LNP was used to deliver FAH mRNA via intravenous injection, protecting FAH^–/–^ mice from rapid weight loss and reducing liver damage markers. Another research group encapsulated two mRNAs into one LNP platform for the treatment of propionic acidemia/aciduria.^[^ ^185^ ^]^ The condition results from the deficiency of propionyl‐CoA carboxylase (PCC), which is comprised of alpha and beta subunits. Through the dual delivery of mRNAs encoding for each subunit, a significant increase in PCC activity was observed in a Pcca^–/–^ mouse model. Additional examples include treatments for arginase deficiency,^[^ ^20^ ^]^ alpha 1‐antitrypsin deficiency,^[^ ^186^ ^]^ citrin deficiency,^[^ ^187^ ^]^ methylmalonic acidemia,^[^ ^188^ ^]^ and ornithine transcarbamylase deficiency.^[^ ^189^ ^]^

Blood‐related genetic diseases are another area in which encouraging results have been achieved using mRNA nanomedicines, which can be used to promote the secretion of therapeutic proteins into circulation. Hemophilia, where clotting factors that are required to stop bleeding are not present in a sufficient quantity, has been a prime target.^[^ ^15^, ^190^, ^191^ ^]^ In one example, an LNP platform was used to deliver human factor IX mRNA for the treatment of hemophilia B.^[^ ^15^ ^]^ After repeated dosing into mice, higher circulating factor IX concentrations, along with higher clotting activity, were achieved. A similar approach was also applied toward the treatment of hemophilia A through the delivery of human factor VIII mRNA.^[^ ^190^ ^]^ In this case, researchers were able to repeatedly increase factor VIII activity levels in a mouse model. Beyond hemophilia, an mRNA‐based nanomedicine solution was also investigated for the treatment of thrombotic thrombocytopenic purpura, which is a potentially fatal disease caused by a deficiency of the ADAMTS13 enzyme.^[^ ^192^ ^]^ Researchers developed an LNP mRNA platform that could produce a wild‐type version of ADAMTS13 or a variant of the enzyme that is resistant to autoantibody induction. Delivery of the variant enzyme mRNA resulted in significantly improved ADAMTS13 activity compared to controls in a knockout mouse model.

mRNA‐based nanomedicine treatments are under development for the treatment of Alzheimer's disease, which can carry a significant genetic component.^[^ ^193^, ^194^ ^]^ This disease is particularly difficult to treat due to the blood‐brain barrier, which can significantly impede the entry of therapeutics into the brain from circulation.^[^ ^195^ ^]^ One treatment method utilized a PEG‐based block catiomer for the mRNA‐based delivery of a single‐chain variable fragment (scFv) against amyloid‐beta (Ab).^[^ ^196^ ^]^ In this case, direct intracranial injection was employed to maximize the production of scFv in the brain for the disaggregation of Ab fibrils. The platform was successful in decreasing Ab burden in an acute amyloidosis mouse model, but it failed to show significant benefits in a transgenic mouse model of Alzheimer's disease. In another study, a cationic polymer‐based PEGylated nanomicelle mRNA formulation was reported to upregulate neprilysin on the surface of neurons for the degradation of Ab.^[^ ^197^ ^]^ Upon intracerebral administration, it was confirmed that significant neprilysin expression and activity could be induced on neuron cells in vivo. This resulted in the significant reduction of synthetic Ab40 that was exogenously administered 24 h after treatment.

Protein replacement therapy using mRNA nanomedicine has also been applied to other rare genetic diseases. A prominent example of this strategy was for the treatment of acute intermittent porphyria (AIP), which is a genetic disease that can sporadically cause a host of complications ranging from loss of appetite to neurovisceral attacks.^[^ ^198^ ^]^ The disease emerges from a lack of porphobilinogen deaminase (PBGD), leading researchers to use LNPs to deliver mRNA encoding for the missing protein. It was found that a 0.5 mg/kg dose of human PBGD mRNA quickly elevated levels of the protein in the liver tissue of mice, and the PBGD remained detectable over the course of 2 weeks. Therapeutic effects were also achieved in a rabbit model of AIP and a mouse model of repeat AIP, where the mice were subjected to three attacks spaced out over a 32‐day period. In non‐human primates, the nanoformulation was able to safely elevate PBGD activity levels relative to baseline for single and multiple dose injection strategies. Besides AIP, mRNA nanomedicines have also demonstrated promise for treating genetic conditions such as cystic fibrosis and Fabry disease.^[^ ^199^, ^200^ ^]^

## 5 CONCLUSION AND OUTLOOK

Significant progress has been made in mRNA medicine in the past several decades, and recent clinical successes have provided additional momentum that will continue to push the field forward. Compared with traditional vaccination and therapeutic platforms, mRNA offers several key advantages. These include the ability to produce almost any protein of interest, allowing for application across a wide range of disease states. By acting in the cytosol, mRNA does not carry the risk of genomic alteration and can be rapidly translated into its protein product. Upon identification of the appropriate genetic sequence, mRNA can be rapidly manufactured and deployed, enabling medical professionals to quickly combat disease outbreaks. Over time, researchers have identified modifications that increase protein translation and reduce unwanted immune responses. By leveraging advancements in nanomedicine, mRNA can be formulated such that it is protected during transit and can be more effectively delivered to target sites. Emerging strategies for enhancing cytosolic delivery can also ensure that mRNA payloads are effectively translated into proteins. Overall, this has resulted in the development of mRNA nanoformulations that have demonstrated preclinical and/or clinical successes against an extensive list of viral infections, cancers, and genetic diseases.

Looking forward, the field will continue to benefit from the development of novel mRNA delivery vehicles with improved biocompatibility, precise targeting, efficient cytosolic localization, and prolonged stability. Along these lines, biomimetic nanoparticles have demonstrated considerable promise, and more sophisticated engineering strategies will enable these platforms to be finely tuned for specific biomedical applications. While current LNP‐based formulations have facile production workflows that are amendable to GMP‐compliant manufacturing, more work is required to verify the scalability of next‐generation mRNA delivery vehicles. Their clinical translation will undoubtedly require the establishment of new approaches for purification as well as quality control, and priority should be given to platforms that are both effective and simple to manufacture. Besides more accurate nanoparticle delivery, the discovery of new mRNA modification strategies that result in more precise translational control can also help to limit off‐target effects. Regarding long‐term stability, it is imperative that future mRNA nanomedicine platforms can be stored without freezing, which will greatly improve their accessibility, particularly in underdeveloped regions of the world. While this represents a challenging problem, future nanoformulation strategies, including the use of novel stabilizer excipients, may provide viable solutions. Overall, the continued advancement of mRNA nanomedicine will help to drive the next wave of clinical adoptions, leading to widespread use and positively impacting human health.

## CONFLICT OF INTEREST

The authors declare no conflict of interest.
